# Analysis of factors affecting the therapeutic effect of propranolol for infantile haemangioma of the head and neck

**DOI:** 10.1038/s41598-017-00495-z

**Published:** 2017-03-23

**Authors:** Jian-Yong Dong, Jie-Xin Ning, Kai Li, Chao Liu, Xu-Xia Wang, Rong-Hui Li, Lin-Lin Yue, Ying-Ying Huang, Shao-Hua Liu

**Affiliations:** 1 Department of Oral and Maxillofacial Surgery, Qilu Hospital, and Institute of Stomatology, Shandong University, Jinan, 250012 China; 2 School of Stomatology, Shandong University, Jinan, 250012 China; 3 School of Medicine, Shandong University, Jinan, 250012 China; 4 Department of Oral and Maxillofacial Surgery, Tianjin Stomatological Hospital, Tianjin, 300041 China

## Abstract

Infantile haemangiomas (IHs) are the most common congenital vascular tumours of infancy. Propranolol has been demonstrated to be effective for IHs; however, the factors affecting its therapeutic effect remain unknown. We enrolled 169 infants with IHs of the head and neck region treated with oral propranolol at a dose of 2.0 mg/kg/day. We evaluated the therapeutic responses 6 months after treatment and the end of treatment, which were categorized into four grades. The type and location of the lesions and the infant age at treatment initiation were analysed. The clinical response rate (III + IV) was 91.72% at 6 months after treatment and 97.63% at the end of treatment. The average treatment duration was 9.99 (2–24) months. The group aged 4–6 months exhibited a greater therapeutic response rate (98.48%). The treatment duration was shorter (9.52 months) for mixed-type IHs. Better therapeutic responses were observed for IHs located around the parotid, periorbital, cheek, and neck regions and for multiple IH lesions. Our study indicated that propranolol is effective for IHs affecting the head and neck. The age at treatment initiation and the location of the lesions had a significant effect on the therapeutic response, whereas the lesion type might affect the treatment duration.

## Introduction

Infantile haemangiomas (IHs) are the most common vascular tumours in infants with a prevalence as high as 10%^1^, approximately 60% of which are located on the head and neck^2^. IHs may cause many complications, particularly during their proliferative phase, including ulceration, visual impairment, and airway obstruction^3^. Following the report by Leaute-Labreze C *et al*. in 2008 indicating that propranolol may treat IHs effectively^4^, propranolol has increasingly become the first-line agent for the treatment of IHs.

Propranolol is generally effective for IHs on the head and neck^5, 6^. However, reports have indicated that propranolol is not effective for all IHs, with a failure rate as high as 10%^7^. In our clinical experience of treating IHs with oral propranolol, the therapeutic response and treatment duration have varied widely according to the different locations of the lesions involving the head and neck. Expectations regarding the therapeutic response and treatment duration are extremely important for clinicians and the parental guardians of the infants.

In this study, we report our clinical observations of head and neck IHs treated with oral propranolol and an analysis of the factors affecting the therapeutic effect.

## Infants and Methods

### Inclusion and exclusion criteria

This study was conducted in the Department of Oral and Maxillofacial Surgery of the Qilu Hospital, Shandong University, China from June 2009 to November 2016. Approval for this study was obtained by the institutional ethics committee of Qilu Hospital, Shandong University. The inclusion criteria were as follows: IHs diagnosed by a medical history and physical examination according to the International Society for the Study of Vascular Anomalies (ISSVA) Classification of Vascular Anomalies^8^ and ultrasonographic or MRI examination. None of the infants were previously treated with other medications. The exclusion criteria were a history or risk of asthma, reactive airway disease, impaired renal or liver function, heart defects or arrhythmias, hypotension, hypersensitivity to propranolol, and lesions involving other regions in addition to the head and neck. Infants who did not adhere to the treatments or follow-up visits were also excluded from the study. The study followed the tenets of the Declaration of Helsinki for research involving human subjects, and informed consent was obtained from all parents whose infants participated in the study. The methods were performed in accordance with the approved guidelines (http://jama.jamanetwork.com/article.aspx?articleid=1760318).

### Propranolol treatment protocol

All infants were administered oral propranolol in two divided doses (at 9 am and 2 pm). The initial dosage was determined by the infant age (1–3 months, 1 mg/kg/day; 4–12 months, 1.5 mg/kg/day). All infants underwent electrocardiographic monitoring 30 min prior to the medication administration and 30 min and 60 min after medication delivery during the first three days. If well tolerated over 2 to 3 days of observation, the dose was increased up to 2.0 mg/kg/day prior to hospital discharge, after which the patient was followed-up at an outpatient clinic every 2 months. The propranolol therapy was stopped when the lesions had completely regressed or when the lesions remained stable for longer than 2 months after at least 1 year of treatment. No other treatment was adopted during the oral propranolol course in our study.

### Clinical evaluation

The following infant data were collected: gender, type and location of lesions, and age at therapy initiation and end dates. The infants were divided into four groups (0–3 months, 4–6 months, 7–9 months and 10–12 months) according to their age at the initiation of treatment. The treatment efficacy was evaluated 6 months after treatment initiation to determine the factors affecting the therapeutic effect of propranolol on IHs of the head and neck. We also evaluated the treatment efficacy at the end of treatment, which was based on the following three tumour-associated parameters: (a) volume reduction; (b) colour improvement; and (c) texture improvement. The evaluations were based on the photographic, ultrasonographic or MR images obtained before and after treatment. The results were categorized into four grades based on the Achauer system^9^: grade I, poor response (0–25% regression); grade II, fair response (26–50% regression); grade III, good response (51–75% regression); and grade IV, excellent response (76–100% regression). We considered grades III and IV indicative of clinical response.

### Statistical analysis

Statistical analyses were performed using standard statistical software packages (SPSS, version20.0, Chicago). Data were expressed as numbers, percentages, or the means ± standard deviations. One-way analysis of variance (ANOVA) was used for the comparisons of treatment duration among different groups. The *Kruskal-Wallis* test was used for the comparison of clinical response rates among the different groups, and a p-value of 0.05 was considered statistically significant.

## Results

### Infants and IH characteristics

A total of 169 infants with head and neck IHs were enrolled in this study. Among the infants, 66 (39%) were male and 103 (61%) were female. Regarding type, mixed haemangioma was predominant, observed in 130 infants, whereas 21 tumours were deep and the remaining 18 were superficial. The most frequently involved head and neck sites were the parotid and perioral regions. A total of 16 patients had multifocal head and neck haemangiomas involving more than one anatomic site. The infants were begun on treatment at an average age of 3.85 months (0.5–11.4 months). The main clinical data are presented in Table 1.

**Table 1:** The clinical data of infants with head and neck infantile haemangioma.

Characteristic	Number	Percentage
Gender (n)		
Male	66	39.05%
Female	103	60.95%
Age at initiation of treatment (n)		
1–3 months	73	43.20%
4–6 months	66	39.05%
7–9 months	22	13.02%
10–12 months	8	4.73%
Average (months)	3.85	
Type of IH (n)		
Superficial	18	10.65%
Deep	21	12.43%
Mixed	130	76.92%
Location of IH (n)		
Parotid	26	15.38%
Perioral	24	14.20%
Periorbital	17	10.06%
Perinasal	18	10.65%
Oral mucosa	16	9.47%
Cheek	12	7.10%
Cranium	13	7.69%
Periauricular	12	7.10%
Forehead	7	4.14%
Neck	5	2.96%
Zygoma	2	1.18%
Occiput	1	0.59%
Multiple	16	9.47%

### Therapeutic effect of oral propranolol for IH

All infants exhibited immediate IH colour lightening, reduced tumour growth rates and obvious softening in texture during hospitalization. Clinical response (grades III + IV) regression rates were observed in 97.63% of the infants at the end of treatment at a dose of 2.0 mg/kg/day. The average treatment duration was 9.99 months (2–24 months).

The results of the therapeutic response evaluations 6 months after treatment initiation were as follows: grade I in 4 infants (2.37%), grade II in 10 infants (5.92%), grade III in 37 infants (21.89%), and grade IV in 118 infants (69.82%). The clinical response rate was 91.72%.

### Effects of age at treatment initiation and lesion type and location on therapeutic effect

Table 2 presents the IH therapeutic responses and treatment durations.

**Table 2:** The infantile haemangioma therapeutic response and treatment duration.

	Clinical response		p-value	Treatment duration	p-value
	III + IV	Rate		$\bar{{\boldsymbol{X}}}{\boldsymbol{\pm }}{\boldsymbol{S}}$ (months)	
Age (months)			0.030		0.375
1–3	62	84.93%		10.58 ± 4.55	
4–6	65	98.48%		9.89 ± 4.97	
7–9	21	95.45%		8.59 ± 4.97	
10–12	7	87.50%		9.25 ± 4.61	
Type			0.716		0.033
Superficial	17	94.44%		10.50 ± 5.01	
Deep	20	95.24%		12.43 ± 3.99	
Mixed	118	90.77%		9.52 ± 4.79	
Location			0.020		0.284
Parotid	26	100.00%		10.92 ± 4.49	
Perioral	22	91.67%		10.21 ± 4.84	
Periorbital	17	100.00%		9.06 ± 5.67	
Perinasal	13	72.22%		9.83 ± 4.07	
Oral mucosa	15	93.75%		11.56 ± 5.21	
Cheek	12	100.00%		10.50 ± 4.94	
Cranium	11	84.62%		6.77 ± 2.22	
Periauricular	10	83.33%		11.00 ± 6.19	
Forehead	5	71.43%		7.57 ± 3.06	
Neck	5	100.00%		11.60 ± 3.0	
Zygoma	2	100.00%		8, 12	
Occiput	1	100.00%		4	
Multiple	16	100.00%		10.13 ± 4.39	
Total	155	91.72%		9.99 ± 4.82	

An analysis by age at treatment initiation revealed that the infants in whom treatment was initiated earlier than 3 months of age had the poorest therapeutic responses and longest treatment durations. This group demonstrated an 84.93% clinical response rate and average treatment duration of 10.58 months. In contrast, the group aged 4–6 months had a 98.48% clinical response rate while the treatment duration for the group aged 7–9 months was 8.59 months. Although the clinical response rate differences among the four groups were significant (p = 0.030) for the initiation of treatment, the treatment duration differences were not significant (p = 0.375).

Regarding the haemangioma type, deep lesions had a superior therapeutic response (95.45%) than mixed (90.77%) and superficial lesions (94.44%), which, although interesting, were not significant findings (p = 0.716). The lesion type had a significant impact on the treatment duration (p = 0.033). The treatment duration for mixed lesions (9.52 months) was reduced compared with deep lesions (12.43 months), and the difference was significant (p = 0.010).

The location of the IHs had a significant impact on the therapeutic response (p = 0.020). Although the therapeutic response of parotid, periorbital, cheek, neck and multiple lesions reached 100%, perinasal (72.22%) and forehead (71.43%) lesions exhibited a poor response to propranolol. The treatment duration of IHs in different locations varied widely (cranium: 6.77 months; neck: 11.60 months); however, these differences were not significant (p = 0.284).

## Discussion

Propranolol is a non-selective beta-adrenergic receptor (β-AR) blocker that has increasingly become an important treatment agent for IHs^10^. To date, no universally accepted protocol exists for the initiation and dosing of propranolol. A prospective study^11^ revealed that propranolol (2.0 mg/kg/day) was effective in Chinese infants with IH. In this study, 2.0 mg/kg/day was used as the regular dose. The clinical evaluation was performed 6 months after oral propranolol administration to assess the factors affecting the therapeutic effect. Grade IV response rates were achieved in 69.82% of infants 6 months after treatment initiation and in 88.76% of infants at the end of treatment, which were higher rates than those reported in other studies^12, 13^. Considering that lesions of the head and neck reportedly have better therapeutic responses to propranolol after an average treatment of 10.5 months^6^, a possible reason for our increased response rates may be that the lesions of the infants enrolled in our study were all located on the head and neck, whereas the lesions in other studies occurred all over the body.

Lesion location was a significant factor affecting the therapeutic response of IHs to propranolol in this study. Previous studies demonstrated that trunk haemangiomas responded poorly and required longer treatment durations than head and neck haemangiomas^6^. In a recent study on pharmacological therapies for IHs^14^, drug treatments for parotid region lesions were reportedly the most efficacious, whereas the least efficacious responses were observed with treatments of the lip region. In our cohort, better responses were observed for haemangiomas of the parotid, periorbital, cheek and neck regions. Lesions in locations where the therapeutic responses have been poor may be treated with other drugs^14^ while those that are not located in cosmetically sensitive areas may be treated with surgery^15^. Among the β-ARs, the β2-AR has a critical role in the action of propranolol^16^. Although no differences were observed in the β2-AR expression levels between four non-responding infants and four matched controls^7^, the differences in β2-AR expression among lesions at different locations and IH stages were not evaluated. We theorize that the effect of location on the therapeutic effect and treatment duration may be due to possible differences in β2-AR expression.

Investigators have observed that children who start treatment earlier have better responses^17, 18^ than older children. In our study, age had a significant impact on the IH treatment duration. The group aged 4–6 months had a higher clinical response rate than the other age groups. The youngest age group (1–3 months) had a poor clinical response, in contrast to previously published results. Additionally, we observed that the younger age groups (1–9 months) required lengthier treatments. We assessed the efficacy of propranolol after 6 months of treatment. The lesions in children who are begun on treatment before 3 months of age may still be in the proliferative phase^19^. Conversely, the lesions in older children may be in the involutional phase. These observations may explain why the younger infants in our study had longer treatment durations and relatively poorer responses. Previous results^18, 20^ have also demonstrated that younger infants require longer treatment than older infants.

IHs are classified into superficial, deep and mixed types according to the depth of the lesions^21^. In this study, deep lesions required significantly lengthier treatments. Because deeper lesions are consistently larger and and have a longer proliferative period^19^, they always require a longer treatment duration. The lesion type had no significant effect on the therapeutic response rates.

The limitations of this study are its retrospective nature, small sample size and high patient variability. Larger prospective studies should be undertaken to investigate the prognostic factors affecting the therapeutic effects of propranolol on infantile haemangiomas of the head and neck.

## References

[1] Haggstrom AN (2007). Prospective study of infantile hemangiomas: demographic, prenatal, and perinatal characteristics. The Journal of pediatrics.

[2] Haggstrom AN, Lammer EJ, Schneider RA, Marcucio R, Frieden IJ (2006). Patterns of infantile hemangiomas: new clues to hemangioma pathogenesis and embryonic facial development. Pediatrics.

[3] Gontijo B (2014). Complications of infantile hemangiomas. Clinics in dermatology.

[4] Leaute-Labreze C (2008). Propranolol for severe hemangiomas of infancy. The New England journal of medicine.

[5] Fuchsmann C (2011). Propranolol as first-line treatment of head and neck hemangiomas. Archives of otolaryngology–head & neck surgery.

[6] Castaneda S (2016). Therapeutic Effect of Propranolol in Mexican Patients with Infantile Hemangioma. Drugs - real world outcomes.

[7] Phillips RJ, Lokmic Z, Crock CM, Penington A (2014). Infantile haemangiomas that failed treatment with propranolol: clinical and histopathological features. Journal of paediatrics and child health.

[8] Enjolras O, Mulliken JB (1997). Vascular tumors and vascular malformations (new issues). Adv. Dermatol..

[9] Achauer BM, Chang CJ, Vander Kam VM (1997). Management of hemangioma of infancy: review of 245 patients. Plastic and reconstructive surgery.

[10] Hermans DJ, Bauland CG, Zweegers J, van Beynum IM, van der Vleuten CJ (2013). Propranolol in a case series of 174 patients with complicated infantile haemangioma: indications, safety and future directions. The British journal of dermatology.

[11] Chang L (2016). Is Propranolol Safe and Effective for Outpatient Use for Infantile Hemangioma? A Prospective Study of 679 Cases From One Center in China. Ann. Plast. Surg..

[12] Buckmiller LM, Munson PD, Dyamenahalli U, Dai Y, Richter GT (2010). Propranolol for infantile hemangiomas: early experience at a tertiary vascular anomalies center. The Laryngoscope.

[13] Pandey V (2014). Propranolol for infantile haemangiomas: experience from a tertiary center. Journal of cutaneous and aesthetic surgery.

[14] Zhang L, Yuan WE, Zheng JW (2016). Pharmacological therapies for infantile hemangiomas: A clinical study in 853 consecutive patients using a standard treatment algorithm. Scientific reports.

[15] O TM, Scheuermann-Poley C, Tan M, Waner M (2013). Distribution, clinical characteristics, and surgical treatment of lip infantile hemangiomas. JAMA facial plastic surgery.

[16] Ji Y, Chen S, Xu C, Li L, Xiang B (2015). The use of propranolol in the treatment of infantile haemangiomas: an update on potential mechanisms of action. The British journal of dermatology.

[17] Phillips RJ, Penington AJ, Bekhor PS, Crock CM (2012). Use of propranolol for treatment of infantile haemangiomas in an outpatient setting. Journal of paediatrics and child health.

[18] Andersen IG, Rechnitzer C, Charabi B (2014). Effectiveness of propanolol for treatment of infantile haemangioma. Danish medical journal.

[19] Chang LC (2008). Growth characteristics of infantile hemangiomas: implications for management. Pediatrics.

[20] Schupp CJ, Kleber JB, Gunther P, Holland-Cunz S (2011). Propranolol therapy in 55 infants with infantile hemangioma: dosage, duration, adverse effects, and outcome. Pediatric dermatology.

[21] Chiller KG, Passaro D, Frieden IJ (2002). Hemangiomas of infancy: clinical characteristics, morphologic subtypes, and their relationship to race, ethnicity, and sex. Arch. Dermatol..

